# Auditory discomfort in visually sensitive individuals

**DOI:** 10.3389/fpsyg.2023.1126481

**Published:** 2023-11-30

**Authors:** Sarah M. Haigh, Anna M. Haugland, Lourdes R. Mendoza, Mackenzie Montero

**Affiliations:** Department of Psychology and Institute for Neuroscience, University of Nevada, Reno, Reno, NV, United States

**Keywords:** auditory, discomfort, pattern glare, sensitivity, frequency

## Abstract

**Introduction:**

Sensory discomfort occurs in clinical and non-clinical populations. While some of the parameters that evoke visual discomfort have been identified, the parameters of sounds that evoke auditory discomfort are largely unknown.

**Methods:**

We presented various sounds and asked participants to rate the discomfort they experienced. In Experiments 1 and 2 tones were presented at frequencies between 0.25-8 kHz and modulated sinusoidally in amplitude at frequencies between 0-32 Hz. In Experiment 3 tones were swept in frequency from 500 Hz-2 kHz at sweep rates of 5-50 per second. In Experiment 4, sweeps varied in frequency range and central frequency.

**Results:**

Discomfort increased with frequency. The effects of the amplitude modulation and sweep rate on discomfort were relatively small and were experienced mainly at low modulation frequencies and high sweep rates. Individuals who experienced visuo-perceptual distortions in the Pattern Glare (PG) Test reported greater auditory discomfort.

**Discussion:**

This suggests that sensory sensitivity in one modality may occur in another.

## 1 Introduction

Discomfort from the sensory environment has been well documented in the visual domain and is associated with a range of clinical symptoms including headaches and, in patients with photosensitive epilepsy, seizures (Wilkins et al., 1984). However, the parameters of auditory stimuli that are uncomfortable to listen to are less well understood. This is despite several clinical conditions being associated with auditory sensitivity, including migraine, autism, and traumatic brain injury.

Scenes from nature have particular spatial (Fernandez and Wilkins, 2008; Juricevic et al., 2010), chromatic (Haigh et al., 2013, 2019; Penacchio et al., 2021), and temporal characteristics (Yoshimoto et al., 2017, 2020). Scenes that do not share these characteristics are generally rated as uncomfortable to look at. Images that are uncomfortable to view for neurotypical viewers tend to be even more uncomfortable for those who are visually sensitive, such as individuals with migraine and those with visual stress, as assessed with the Pattern Glare Test. This suggests that there may be a continuum of sensory sensitivity across the population with certain clinical conditions at the extreme of the spectrum.

In the auditory domain, unnatural sounds (e.g., engines and alarms) are typically found to be unpleasant, whereas more natural sounds (leaves rustling and birdsong) are more pleasant (Axelsson et al., 2010). This suggests there are preferences for natural sounds similar to preferences to natural visual images. It is important to note that “pleasantness” is used more often in the auditory literature and “discomfort” in the visual literature.

When assessing the spectral and temporal properties of common sounds that are unpleasant to listen to, for example, chalk on a chalkboard, carrier frequencies (that convey the overall pitch of the sound) between 2.5–5.5 kHz and 1–16 Hz modulation frequencies (that can add “texture” or “wobble” to a sound) were identified as common factors (Kumar et al., 2008). However, there is some evidence to suggest that knowing the source of the unpleasant sounds such as chalk on a chalkboard can make sounds even more unpleasant (Reuter and Oehler, 2011). Natural environmental sounds and speech have specific pitch and loudness properties and these properties can predict human auditory psychophysical thresholds to some extent (Monson et al., 2013). Furthermore, recent experience with sounds of certain pitch or loudness can shift our perception of new stimuli (Han et al., 2011). Together, these findings imply that discomfort and unpleasantness may depend on which part(s) of the auditory system are active when encoding the information – the cochlear or brainstem that are sensitive to physical properties such as pitch or loudness (as modeled by Shamma, 2003) or later areas that encode the semantic properties of the sound.

It is possible that those who experience increased discomfort in one sensory modality also experience discomfort in other modalities. For example, individuals with migraine report auditory sensitivity during and between migraine attacks (Main et al., 1997), and photophobia and phonophobia are included in the criteria for migraine diagnosis (International Headache Society [IHS], 2018). Ratings of unpleasantness appear to be greater for migraine patients when listening to unnatural sounds (sirens) compared to natural sounds (waves; Ishikawa et al., 2019), as in the visual domain. Although the diagnosis of migraine places equal emphasis on photophobia and phonophobia, measures of neural connectivity (coherence across EEG electrodes) has shown greater connectivity over visual areas in migraine. Interestingly, the pattern of activation was similar during visual and during auditory stimulation (steady-state evoked potentials; Chamanzar et al., 2021). Therefore, in the current study, we identified individuals who experience visual sensitivity to identify if they are more likely to be auditorily sensitive too.

For this series of experiments, we had two aims. The first aim was to identify some of the parameters of auditory stimuli that evoke discomfort. To do this, we chose simple auditory stimuli where we could systematically vary one or two parameters independently to identify their effect on discomfort. We drew inspiration from the parameters that evoke visual discomfort such as spatial frequency, color, and flicker.

In Experiments 1 and 2, we chose amplitude modulated (AM) tones so we could vary the carrier and modulation frequencies, which follows on from the findings reported by Kumar et al. (2008). AM tones are arguably analogous to spatial frequency and color which impact visual discomfort. The carrier frequencies in these sounds conveyed the overall pitch or the sound. The modulation frequencies added “wobble” to the sounds so that the higher the modulation frequency, the more “wobble” there was in the sound. In Experiment 3, we manipulated how may frequency sweeps were presented in a second. The repetitious nature of the sweeps is analogous to visual flicker, which is also noxious to the visual system, although at much higher rates than we tested here. Experiment 4 presented frequency sweeps that varied in the central frequency and the range of frequencies covered in the sweeps. While AM tones and frequency sweeps are rarely encountered in daily life by themselves, the individual parameters are present in a lot of audio media. Identifying the simple parameters of the stimuli that drive discomfort will provide targets to avoid when developing and improving media and technology.

The second aim was to identify if there were individual differences in auditory discomfort. Owing to the growing literature on sensory sensitivities in migraine and the inclusion of phonophobia as a symptom in the diagnosis of migraine (International Headache Society [IHS], 2018), we compared individuals with migraine to headache-free individuals. However, not all individuals with migraine experience phonophobia (Choi et al., 2009). Therefore, we also included the Pattern Glare Test in our protocol (Experiments 2–4) to identify those who were visually sensitive regardless of whether they had migraines or not. The Pattern Glare Test measures the number of visual illusions seen in a striped achromatic pattern, such as seeing the stripes flicker, shimmer, or disappear. The more illusions they see, the more visually sensitive they are (Wilkins et al., 1984). Those who saw greater than two different types of illusions were in the high pattern glare group. Those who saw few or no illusions were in the low pattern glare group. From here, we can ascertain if those with high pattern glare/who are visually sensitive are more auditorily sensitive too. If so, then this would suggest a domain general mechanism for sensory discomfort.

## 2 Experiment 1: impact of amplitude modulation on auditory discomfort

Experiment 1 assessed ratings of discomfort to pure tones that differed in pitch and were modulated at different frequencies. To identify potential individual differences in auditory discomfort, we then compared individuals with migraine to those who were headache-free. Migraine includes phonophobia as one of the key symptoms in the diagnosis (International Headache Society [IHS], 2018) and so we predicted that those with migraine would rate the sounds as being more uncomfortable to listen to than headache-free individuals.

### 2.1 Methods

#### 2.1.1 Participants

The study was conducted online (Qualtrics) for one semester (16 weeks). An *a priori* power analysis was conducted using effect sizes from the visual discomfort literature (Haigh et al., 2019) using G*Power (Faul et al., 2007, 2009). To be able to detect a medium-to-large effect size (*d* = 0.8) between migraine and headache-free individuals, we would need a minimum of 44 participants.

Sixty-two participants took part (48 female, 14 male; mean age 19.9 years old, SD 5 years; four participants declined to provide their age) from the University of Nevada, Reno student population. None of the participants had epilepsy or a history of seizures. None reported a psychological or neurological diagnosis or were on any medications for psychological conditions. We also actively recruited individuals with migraine and headache-free individuals. All participants completed a headache questionnaire based off the International Headache Society (IHS) criteria for migraine. Twenty-nine satisfied the IHS criteria for migraine (27 female, 2 male; mean age 18.8 years, SD 1.8 years) and 26 were headache-free (17 female, 9 male; mean age 21 years old, SD 7.0 years). The age and gender breakdown of all participants who took part in all four experiments is shown in Table 1. Participants gave electronic assent via Qualtrics before beginning the study. No identifiable information was collected except for name and email if the participant chose to enter a raffle for a $10 Amazon gift card. All participants received course credit upon completion of the experiment.

**TABLE 1:** Gender (female and male) and mean age of participants in each experiment, shown separately for the migraine, headache-free, high pattern glare (PG), and low PG groups.

	Experiment 1	Experiment 2	Experiment 3	Experiment 4
Total – gender	F48/M14	F11/M8	F51/M18/1 declined	F34/M28
Age (SD), years	19.9 (5)	19.6 (2.1)	19.6 (1.7)	19.8 (1.4)
Migraine	F27/M2	N/A	F18/M7	F9/M6
Age (SD), years	18.8 (1.8)	N/A	19.9 (1.5)	20 (1)
Headache-free	F17/M9	N/A	F8/M4/1 declined	F10/M12
Age (SD), years	21 (7)	N/A	19 (1.4)	19.5 (1.6)
High PG	N/A	F6/M5	F27/M8	F19/M7
Age (SD), years	N/A	18.8 (1.8)	19.7 (1.9)	19.8 (1.5)
Low PG	N/A	F5/M3	F19/M8/1 declined	F15/M21
Age (SD), years	N/A	20.5 (2.5)	19.4 (1.5)	19.9 (1.4)

These studies were conducted in accordance with the ethical standards of the 1964 Helsinki Declaration. All protocols were approved by the Institutional Review Board at the University of Nevada, Reno (28/01/2019; ID number 1333057). Informed consent was obtained from all individual participants included in the study.

We also provided participants a hyperacusis questionnaire to complete in Qualtrics after completing the ratings (Khalfa et al., 2002). The aim here was to identify participants who were auditory sensitive independent of migraine diagnosis. However, when the responses to the questions were summed, only two participants had summed scores greater than 28, which was the cutoff for having hyperacusis (according to Khalfa et al., 2002). Therefore, we did not include the hyperacusis measure in the analyses.

#### 2.1.2 Stimuli

All sounds used in this study are available on OSF^1^ along with the MATLAB (Mathworks) scripts used to generate the sounds.

All sounds were sampled at 44,100 Hz with 16-bit resolution and saved as wav files to upload to Qualtrics. Pure tone sinusoidal sounds at (carrier) frequencies of 0.25, 0.5, 1, 1.5, 2, 3, 4, 6, and 8 kHz (conveying overall pitch) were presented for 1 s and were sinusoidally modulated at 100% by a 0, 2, 4, 8, 16, or 32 Hz frequency (generating “wobble” in the sounds). Each tone contained a 5 ms ramp up and ramp down to avoid transducer “clicks” from the speakers. Due to the known perceptual link between pitch and loudness, the “acousticLoudness” MATLAB function was used to estimate perceived loudness (ISO 523-1:2017; ISO 532-2:2017) and adjust the intensity of the tone accordingly. While this method does not exclude the potential confound of any discomfort from carrier frequency being partially due to increased loudness, it prevented participants from needing to adjust the volume of their head/earphones during the study.

#### 2.1.3 Procedure

Tones were presented over the Qualtrics online survey software. Participants were required to wear headphones or earphones and to complete the study on a laptop or desktop computer in a quiet environment. Each tone was presented three times in a random order. Once the tone finished playing, the participant was asked to rate how uncomfortable it was to listen to on a scale from 0 to 10 where “0” was “Fine to listen to,” “5” was “Somewhat uncomfortable,” and “10” was “Extremely uncomfortable.” We have used similar ratings scales previously and have found consistent results across studies (Haigh et al., 2013, 2019; Lindquist et al., 2021). At the end of the study, participants were asked to rate the 0 Hz modulation frequency tones in order of loudness to indicate how different the loudness of the tones was perceived to be.

#### 2.1.4 Data analysis

A mixed-measures ANOVA was conducted with carrier frequency (conveys overall pitch; 0.25, 0.5, 1, 1.5, 2, 3, 4, 6, and 8 kHz) and modulation frequency [adds wobble to the sound; 0 Hz (no wobble), 2, 4, 8, 16, or 32 Hz (lots of wobble)] as repeated-measures factors, and migraine and headache-free individuals as a between-subjects measure. Due to the large gender discrepancy in the sample, we initially included gender in the ANOVA as another between-subjects factor. However, there was no significant effect of gender on discomfort or any interactions with gender, and so this factor was removed from the analyses described below. Separate analyses of the migraine and headache-free groups or of all 62 participants showed the same overall results. Therefore, for simplicity, the analysis of the migraine and headache-free individuals only are described. Any missing ratings of discomfort (1.14%) were accounted for by taking the average rating for that sound (the winsorize method). Follow-up analyses of significant effects were conducted using pairwise Bonferroni corrected *t*-tests. Cohen’s *d* effect size was calculated for the main effects.

### 2.2 Results

**FIGURE 1:**
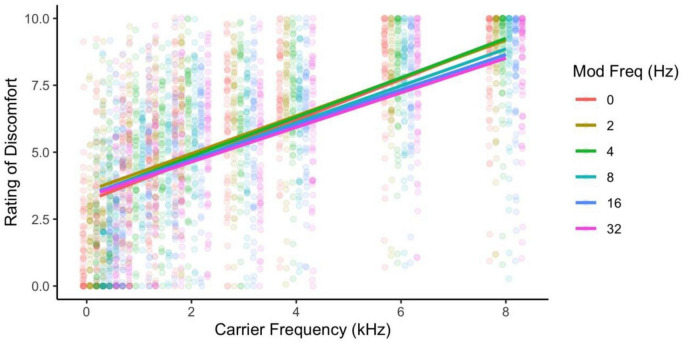
Ratings of discomfort increased with both carrier and modulation frequency, and the higher carrier frequencies showed the larger effects of higher modulation frequency reducing discomfort. Ratings are from the full sample of participants.

**FIGURE 2:**
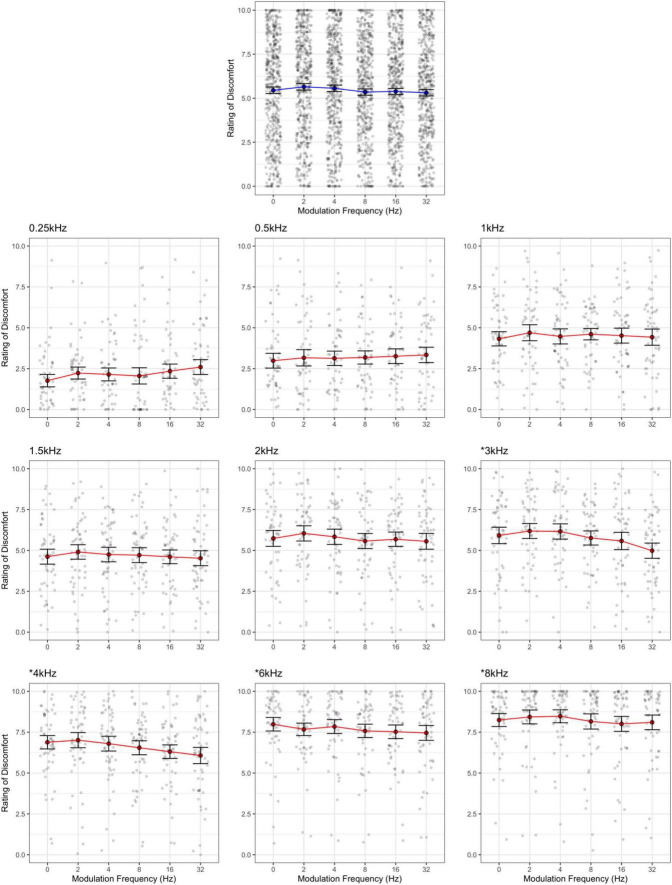
Rating of discomfort for each modulation frequency shown separately for each carrier frequency. Ratings are from the entire sample. Error bars show 3× standard error so that they are visible. Note that the discomfort is lowest for the 0.25 kHz carrier frequency and highest for the 8 kHz carrier, regardless of modulation frequency. The significant interaction was due to carrier frequencies 3, 4, 6, and 8 kHz showing a reduction in discomfort with increasing modulation frequency (highlighted by asterisk).

**FIGURE 3:**
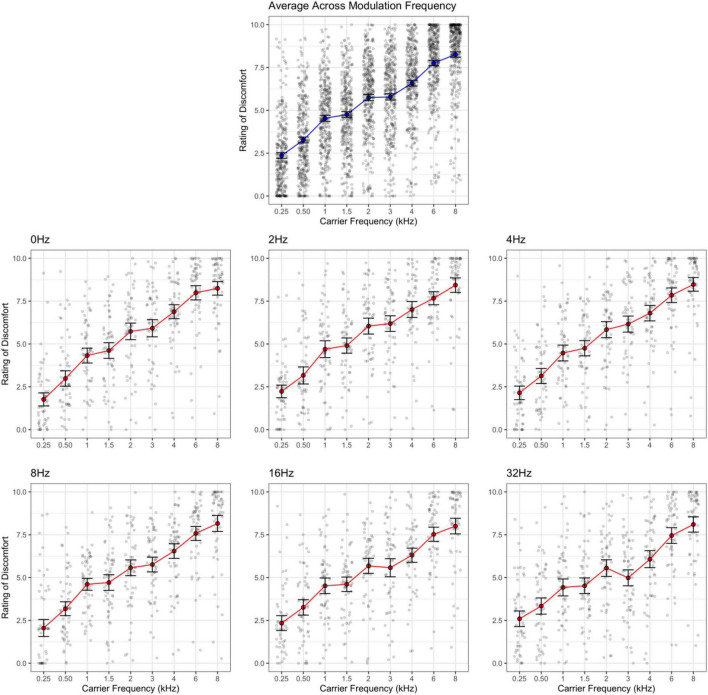
Rating of discomfort for each carrier frequency shown separately for each modulation frequency. Ratings are from the entire sample. Error bars show 3× standard error so that they are visible. Note that the effect of carrier frequency is consistent across all modulation frequencies. The interaction effect is less evident here.

**FIGURE 4:**
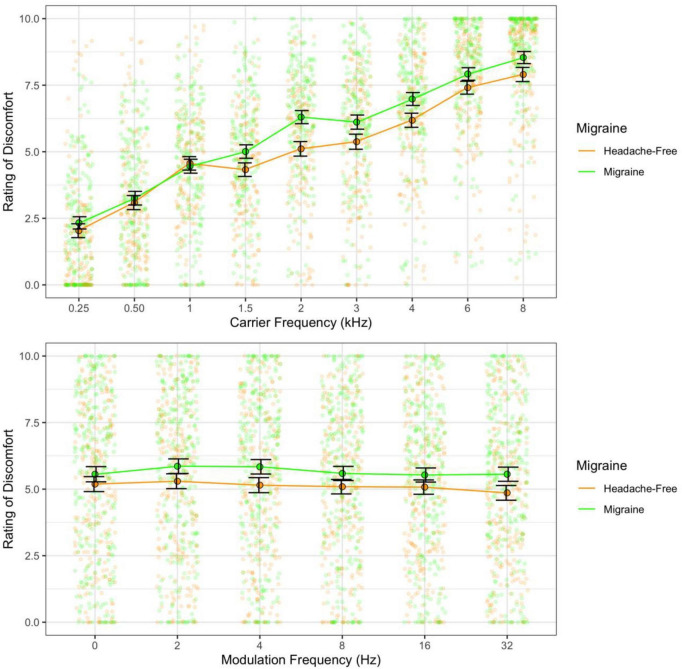
Ratings of discomfort for migraine and headache-free groups for each carrier **(top)** and modulation frequency **(bottom)**. Migraine reported nominally greater discomfort and this is consistent across carrier and modulation frequencies; however, was not significant. Error bars show 3× standard error so that they are visible.

Ratings of discomfort increased significantly with carrier frequency [*F*(8,424) = 145.85, *p* < 0.001; η^2^ = 0.42; *d* < 3.77], and decreased with modulation frequency [*F*(5,265) = 3.76, *p* = 0.003; η^2^ = 0.002; pairwise comparisons, *p* < 0.03; *d* < 0.23], although the pure tone was rated as being more uncomfortable than the 2 Hz modulation (*p* = 0.014; Figure 1). Interestingly there was an interaction between carrier and modulation frequency [*F*(40,2120) = 2.12, *p* < 0.001; η^2^ = 0.005] where the higher carriers (3, 4, 6, and 8 kHz) showed a reduction in discomfort with higher modulation frequencies, but not in the lower carrier frequencies (0.25, 0.5, 1, 2 kHz, *p* < 0.05; best illustrated in Figure 2, also see Figure 3). Surprisingly, while individuals with migraine reported nominally greater discomfort than headache-free individuals, the group differences were not significant [*F*(1,53) = 1.77, *p* = 0.189; *d* = 0.19; η^2^ = 0.009; Figure 4].

## 3 Interim discussion

Auditory discomfort was clearly impacted by both carrier and modulation frequency. Interestingly, the migraine group did not report significantly greater discomfort overall, despite a reliable trend of increased ratings of discomfort. Without measures of visual discomfort to complement, it is difficult to say whether this group of individuals with migraine experienced more or less auditory sensitivity than they would have experienced in an equivalent visual sensitivity task (Marcus and Soso, 1989; Haigh et al., 2012, 2019). For the remaining experiments, we added the Pattern Glare Test to identify whether those with visual sensitivity (independent of having migraine) were more sensitive to certain auditory stimuli. The Pattern Glare Test asks participants to report if they saw any illusions in a striped pattern, and if they did, what the illusions looked like. For example, the stripes are often reported to shimmer, flicker, or change color. The higher the number of illusions the participant reports seeing, the greater their visual sensitivity (Wilkins et al., 1984; Wilkins and Evans, 2001). This will provide a more comprehensive understanding of how uncomfortable individuals with visual sensitivity find certain auditory stimuli.

One caveat from Experiment 1, is that higher carrier frequencies (the pitch) perceptually sound louder. As the experiment was conducted online, the equipment being used varied. This doubtless increased the variance in the data and any emerging trends are likely be stronger under more controlled conditions. Therefore, we conducted a study similar to Experiment 1 but in-person, where all tones were adjusted to have a similar subjective volume before a subset of the sounds were rated for discomfort.

## 4 Experiment 2: impact of amplitude modulation on discomfort when accounting for perceived loudness

To assess whether the effects reported in Experiment 1 were due to the online nature of the stimulus presentation, we conducted the study in-person with a new sample of participants. The main differences were that fewer of the sounds were presented (in order to reduce the experimental time), and we assessed the effect of individual differences in pattern glare as a measure of visual sensitivity. The aim was to identify if those who were visually sensitive were more likely to be more sensitive/report higher ratings of discomfort to the auditory stimuli.

### 4.1 Methods

#### 4.1.1 Participants

Nineteen participants took part (11 female, 8 male; average age 19.6 years, SD 2.1 years; one participant did not provide their age). Participants completed the Pattern Glare Test (Wilkins and Evans, 2001; for details, see below). Eleven participants experienced high pattern (greater than 2 illusions; 6 female, 5 male; mean age 18.8 years old, SD 1.8 years) and eight were categorized as low pattern glare (5 female, 3 male; mean age 20.5 years old, SD 2.5 years). As this was a smaller study than Experiment 1, we did not compare individuals with migraine and headache-free individuals. All participants gave their signed informed consent. None of the participants reported a neurological or psychiatric condition. None of the participants took part in Experiment 1.

#### 4.1.2 Stimuli

A subset of the sounds used in Experiment 1 were used in Experiment 2 to help shorten the testing time. Pure tone sounds at frequencies of 0.5, 1, 2, 4, and 8 kHz were presented for 1 s and were modulated by a 0, 2, 4, 8, 16, or 32 Hz modulation frequency. Each tone contained a 5 ms ramp up and ramp down to avoid transducer clicks.

#### 4.1.3 Pattern Glare Test

**FIGURE 5:**
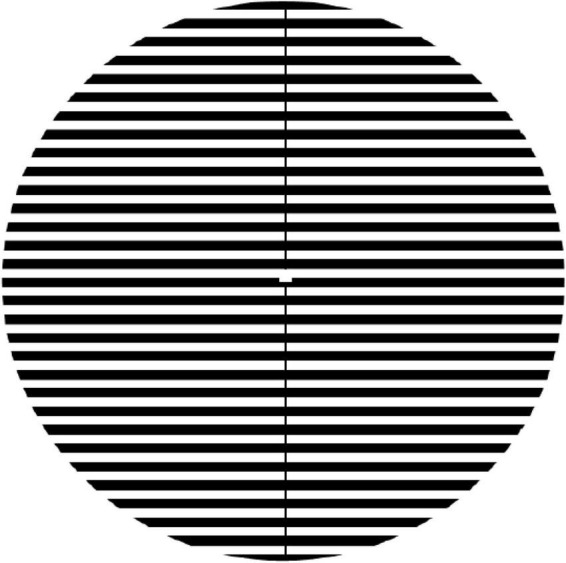
The pattern used for the Pattern Glare Test. For Experiment 2, the pattern was presented at 100% contrast at 3.1cpd. For Experiments 3 and 4, the pattern was presented online (Qualtrics) and so we had no control over the spatial and luminance parameters.

The Pattern Glare Test comprises an achromatic horizontal striped grating pattern presented for 5 s at 3.1 cpd (see Figure 5). Once the pattern had disappeared, participants were asked if they saw any of the following illusions: shadowy shapes amongst the lines, shimmering of the lines, flickering, colors, blur, bending of the lines, or if they experienced any of the following sensations: pain/discomfort, nausea/dizziness, or unease. Participants also had the option to enter in other sensations that they experienced but were not listed. The number of illusions and sensations experienced were summed for each participant.

#### 4.1.4 Equipment

The study was conducted on a Dell laptop and sounds were presented using Etymotic ER2 earphones. All stimuli were generated and presented using MATLAB.

#### 4.1.5 Procedure

The Pattern Glare Test and demographics questionnaire were completed online on Qualtrics. For the Pattern Glare Test, participants were shown the mid spatial frequency grating pattern for 10 s and were then asked to use the published list to select the illusions they saw in the pattern or to indicate if they did not see any, or if they felt any discomfort.

Participants then completed two tasks in person using the same computer and same earphones. The first was to establish the equivalent loudness for each pitch for each participant. Before beginning the tasks, participants were presented with the highest and the lowest pitches to allow them to adjust the volume of the computer so that they can hear both sounds without causing pain. This means that dB of the tones varied across participants but was fixed for the duration of the study.

To establish the loudness levels, participants were presented with the pure tone 8 kHz sound followed by either the 0.5, 1, 3, or 4 kHz pure tone after an ISI of 1 s. Participants then had to respond whether the second tone sounded louder or quieter than the first. If it was louder, then they pressed the down key to reduce the volume, and if it was quieter, then they pressed the up key to increase the loudness. When the loudness was the same, they pressed the “enter” key. Each tone was presented three times in a random order to gain the best estimate of loudness.

For the second task, the same stimulus presentation as described in Experiment 1 was used except presented using MATLAB. Tones were presented for 1 s and participants were asked to rate how uncomfortable the tones were to listen to using a scale from 1 (not uncomfortable) to 9 (very uncomfortable). Participants had unlimited time to make their response. Each tone was presented three times in a random order.

#### 4.1.6 Data analysis

A mixed-measures ANOVA was conducted with carrier frequency (pitch) and modulation frequency (wobble) as repeated-measures and the effect of pattern glare (PG; high and low) was used as the between-subjects factor. *Post hoc* analyses to dissect significant effects were conducted using pairwise Bonferroni corrected *t*-tests. Cohen’s *d* effect size was calculated for the main effects.

### 4.2 Results

**FIGURE 6:**
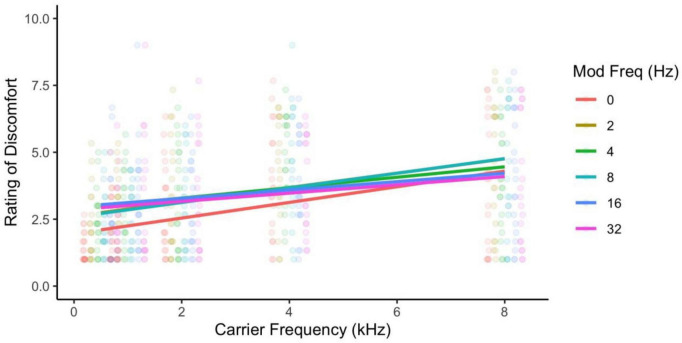
Ratings of discomfort for carrier and modulation frequency. The interaction shows that modulation frequency increased discomfort for the lower carrier frequencies and decreased discomfort for the higher carrier frequencies.

**FIGURE 7:**
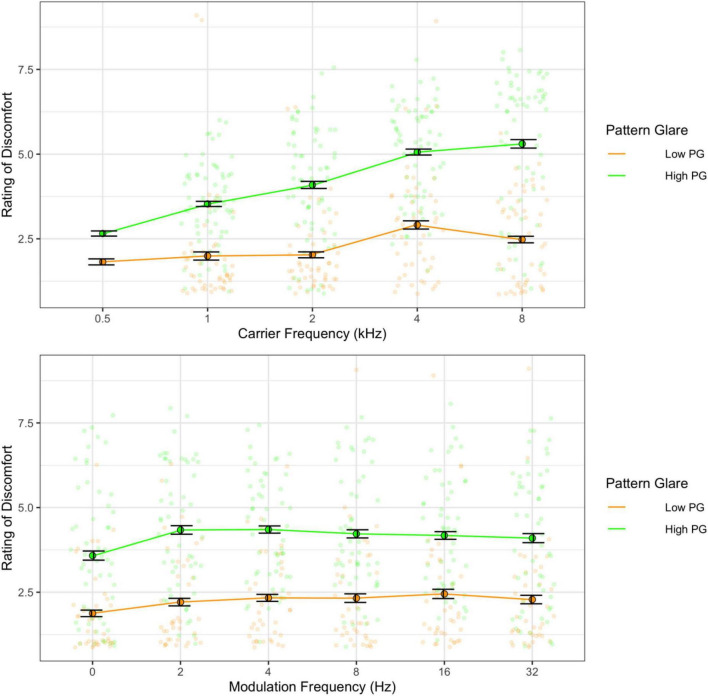
Ratings of discomfort as a function of carrier frequency **(top)** and as a function of modulation frequency **(bottom)** shown separately for the low and the high pattern glare (PG) groups. Error bars show 1 standard error. The high PG group reported consistently greater discomfort for all carrier and modulation frequencies.

As in Experiment 1, there was a main effect of carrier frequency with higher frequencies being perceived as more uncomfortable than low frequencies [*F*(4,68) = 16.93, *p* < 0.001; η^2^ = 0.14; pairwise *t*-tests, *p* < 0.001; *d* < 1.50], and the pure tone was rated as significantly less uncomfortable than the modulated tones [*F*(5,85) = 3.66, *p* = 0.005; η^2^ = 0.01; pairwise comparisons, *p* < 0.001; *d* < 0.50]. Once again, there was a significant interaction [*F*(20,340) = 2.17, *p* = 0.003; η^2^ = 0.01; Figure 6] such that lower carrier frequencies (0.5 and 1 kHz) showed an increase in discomfort with higher modulation frequencies, whereas higher carriers (4 and 8 kHz) showed a decrease in discomfort with higher modulation frequencies (*p* < 0.05; see Supplementary Figures 1, 2). Those with high PG rated the tones as being more uncomfortable than those with low PG [*F*(1,17) = 15.68, *p* = 0.001; η^2^ = 0.23; *d* = 1.11; Figure 7] and reported significantly greater effects of high carrier frequency on discomfort [interaction between PG groups and carrier frequency; *F*(4,68) = 3.55, *p* = 0.011; η^2^ = 0.03].

## 5 Interim discussion

These findings replicate the results from Experiment 1, even when differences in perceived loudness are accounted for. The interaction between carrier and modulation frequency was slightly different from Experiment 1, suggesting that controlling for perceptual effects of pitch on loudness may have helped uncover more subtle effects on discomfort.

In Experiment 1, the auditory discomfort in individuals with migraine did not differ significantly from that in headache-free individuals (*d* = 0.19). In Experiment 2, the high PG group consistently reported greater discomfort compared to the low PG group (*d* = 1.11). Together, this suggests that those who are visually sensitive are likely to experience auditory sensitivity too. The much larger effect size between pattern glare groups compared to migraine and headache-free individuals suggests that auditory sensitivity may not be as prevalent in the migraine population as previously perceived.

In the remaining experiments, we assessed auditory discomfort to frequency sweeps and continued to compare individuals with high and low PG as well as migraine and headache-free individuals to identify if the same pattern of results persists in new participant samples.

## 6 Experiment 3: impact of number of frequency sweeps on auditory discomfort

We next assessed the effect of presentation rate on discomfort using frequency sweeps. Frequency sweeps start the sounds at a fixed frequency and increase (or decrease) the frequency presented until the final frequency within a fixed (often short) time period. We chose to use frequency sweeps because they contain a range of frequencies and so the effects on discomfort are less likely to be frequency-specific (as seen in Experiments 1 and 2). We continued to assess the individual differences in auditory discomfort by comparing migraine and headache-free individuals and comparing the effects of pattern glare (a measure of visual sensitivity). We predicted that individuals with high patten glare would report greater discomfort than those with low pattern glare, similar to the findings in Experiment 2.

### 6.1 Methods

#### 6.1.1 Participants

Seventy participants (51 female, 18 male, one declined; average age 19.6 years, SD 1.7 years; three declined to provide their age). Twenty-five had migraine (according to IHS criteria; 18 female, 7 male; 19.9 years old, SD 1.5 years) and 13 were headache-free (8 female, 4 male, 1 declined; mean age 19 years old, SD 1.4 years). Seven participants did not complete the Pattern Glare Test. Out of the 63 participants, 35 had high PG (reported seeing 2 or more illusions in the Pattern Glare Test; 27 female, 8 male; 19.7 years old, SD 1.9 years) and 27 had low PG (19 female, 8 male, 1 declined; 19.4 years old, SD 1.5 years). The overlap in the number of participants who had migraine and who experienced high pattern glare is reported in Supplementary Table 1.

Participants gave electronic assent via Qualtrics before beginning the study. No identifiable information was collected except for name and email if the participant chose to enter a raffle for a $10 gift card. All participants received course credit upon completion of the experiment. None of the participants took part in Experiments 1 or 2.

#### 6.1.2 Stimuli

The “chirp” function in MATLAB was used to generate frequency sweeps of a given length sampled at 44,100 Hz at 16-bits. Spectrograms were used to verify the timings and frequencies contained within the sweeps. Sweeps increasing in frequency from 500 Hz to 2 kHz were presented 5, 10, 15, 20, 25, 30, 35, 40, 45, or 50 times within a 1 s period with a sawtooth profile. Each sweep contained a 0.5 ms ramp up and ramp down to avoid clicks from the transducer. Each 1-s trial was saved as a wav file and uploaded to Qualtrics to preserve the timings of the sweeps.

#### 6.1.3 Procedure

Similar to Experiment 1, the sounds were presented over the Qualtrics online survey software. Participants were required to wear headphones or earphones and to complete the study on a laptop or desktop computer in a quiet environment. Each trial of frequency sweeps was presented four times in a random order. Once the trial finished playing, the participant was asked to rate how uncomfortable the tone was to listen to on a scale from 0 to 10 where “0” was “Fine to listen to” and “10” was “Extremely uncomfortable.” Participants also completed the Pattern Glare Test on Qualtrics, same as Experiment 2.

#### 6.1.4 Data analysis

A mixed-measures ANOVA was conducted with sweep rate (5, 10, 15, 20, 25, 30, 35, 40, 45, and 50 Hz) as a repeated-measure and the effect of group (high and low PG) was used as the between-subjects factor. Due to the differences in numbers of males and female in the sample, we included gender in the ANOVA as another between-subjects factor. However, similar to Experiment 1, there was no significant effect of gender on discomfort or any interactions with gender, and so this factor was removed from the analyses described below. A separate ANOVA was conducted to compare the migraine and headache-free participants as the between-subject factor too. *Post hoc* analyses to dissect significant effects were conducted using pairwise Bonferroni corrected *t*-tests. Cohen’s *d* effect sizes were calculated for the main effects.

### 6.2 Results

**FIGURE 8:**
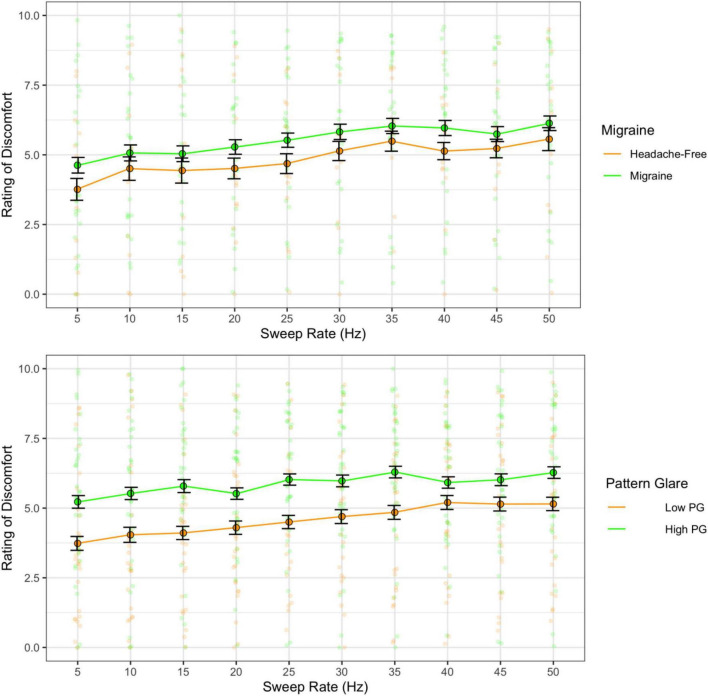
Ratings of discomfort as a function of sweep frequency for the migraine and headache-free groups **(top)** and the high and low pattern glare (PG) groups **(bottom)**. Error bars 1 standard error. Higher sweep rates increased discomfort. Ratings of discomfort were nominally higher in migraine compared to headache-free individuals. However, ratings of discomfort were significantly higher in individuals with high PG compared to low PG regardless of sweep rate.

The higher the rate of the frequency sweeps, the greater the discomfort [*F*(9,324) = 7.20, *p* < 0.001; η^2^ = 0.03; *d* < 1.39], but there were no differences between migraine and headache-free groups [*F*(1,36) = 0.65, *p* = 0.426; η^2^ = 0.01; *d* = 0.25]. However, there was a difference between PG groups, where individuals with high PG reported greater auditory discomfort than the low PG group [*F*(1,61) = 4.85, *p* = 0.031; η^2^ = 0.06; *d* = 0.5; Figure 8].

## 7 Interim discussion

Increasing the sweep rate increased auditory discomfort as did the modulation of AM tones in Experiments 1 and 2. Therefore, it appears that presentation frequency as well as carrier frequency both independently impact auditory discomfort. The findings from Experiment 3 also replicated the small and non-significant increase in discomfort ratings in migraine and the significantly greater auditory discomfort in those with high PG, continuing to support the idea that pattern glare may be useful for identifying those with auditory sensitivity too.

For the final experiment, we focused on the impact the range of frequencies contained within a sweep and the central frequency within a sweep has on discomfort. This offered a clearer picture of the role perceived pitch has on discomfort.

## 8 Experiment 4: impact on discomfort from sweeps

Finally, we assessed the parameters of frequency sweeps that drive discomfort. We manipulated the range of frequencies contained within a sweep, the central frequency of the sweep, the starting frequency, and the end frequency to identify which component(s) were related to auditory discomfort. Similar to Experiment 3, we compared migraine and headache-free individuals, and those with high and low pattern glare to identify individual differences in auditory discomfort. This also serves to replicate the previous findings from Experiments 1–3 in a new sample.

### 8.1 Methods

#### 8.1.1 Participants

Sixty-two participants (34 female, 28 male; average age 19.8 years, SD 1.4 years, 1 declined). Sixteen had migraine (according to IHS criteria; 9 female, 6 male; 20 years old, SD 1 year) and 22 were headache-free (10 female, 12 male; 19.5 years old, SD 1.6 years). Out of the 62 participants, 26 had high PG (reported seeing 2 or more illusions in the Pattern Glare Test; 19 female, 7 male; 19.8 years old, SD 1.5 years) and 36 had low PG (15 female, 21 male; 19.9 years old, SD 1.4 years). The number of participants who had both migraine and experienced high pattern glare is reported in Supplementary Table 1. None of the participants took part in Experiments 1–3.

Participants gave electronic assent via Qualtrics before beginning the study. No identifiable information was collected except for name and email if the participant chose to enter a raffle for a $10 Amazon gift card. All participants received course credit upon completion of the experiment.

#### 8.1.2 Stimuli

The same MATLAB code and verification were used to generate the sweeps for Experiment 4. However, in comparison with Experiment 3, we presented five sweeps for all trials. To assess the effects of central frequency and range of frequencies we designed four categories of frequency sweeps (see Table 2 for summary of sweeps). First, we kept the frequency range the same at 500 Hz and varied the central frequency from 750 to 1,750 Hz in increments of 250 Hz (category 1). Second, we kept the central frequency the same at 1,250 Hz and increase the range (250–1,250 Hz) in 500 Hz increments (category 2). Third, we varied both the central frequency and range by starting the frequency sweep at 500 Hz and increasing the end frequency by 250 Hz increments to 2,000 Hz, so that the range increased from 250 to 1,500 Hz, and central frequency varied (category 3). Finally, we ended the frequency sweeps at 2,000 Hz and decreased the starting frequency from 1,750 Hz by 250 Hz increments to 500 Hz (range increased from 250 to 1,500 Hz and central frequency varied; category 4). Each sweep contained a 5 ms ramp up and ramp down to avoid transducer clicks. Each group of five sweeps was saved as a wav file and uploaded to Qualtrics to maintain the timings of the sweeps.

**TABLE 2:** Start and end frequencies (in Hertz; Hz) for each of the sweeps used.

	Start frequency	End frequency	Central frequency	Range	Duplicate?
Category 1	500	1,000	750	500	
	750	1,250	1,000	500	
	1,000	1,500	1,250	500	Y
	1,250	1,750	1,500	500	
	1,500	2,000	1,750	500	
Category 2	1,125	1,375	1,250	250	
	1,000	1,500	1,250	500	Y
	875	1,625	1,250	750	
	750	1,750	1,250	1,000	
	625	1,875	1,250	1,250	
	500	2,000	1,250	1,500	Y
Category 3	500	750	625	250	
	500	1,000	750	500	
	500	1,250	875	750	
	500	1,500	1,000	1,000	
	500	1,750	1,125	1,250	
	500	2,000	1,250	1,500	Y
Category 4	500	2,000	1,250	1,500	Y
	750	2,000	1,375	1,250	
	1,000	2,000	1,500	1,000	
	1,250	2,000	1,625	750	
	1,500	2,000	1,750	500	
	1,750	2,000	1,875	250	

Central frequency and range of frequencies are calculated. Sweeps that fell into two categories are highlighted in the Duplicate column. These tones were not presented twice as frequently; this is for illustration only.

#### 8.1.3 Procedure

Consistent with Experiment 3, the Pattern Glare Test and the sweeps were presented on Qualtrics. Participants were required to wear headphones or earphones and to complete the study on a laptop or desktop computer in a quiet environment. Each frequency sweep was presented once in a random order. Once the sound finished playing, the participant was asked to rate how uncomfortable it was to listen to the sound on a scale from 0 to 10 where “0” was “Fine to listen to” and “10” was “Extremely uncomfortable.”

#### 8.1.4 Data analysis

Because of the four categories of sweeps used, it was not possible to run a single mixed ANOVA to assess the effects of frequency and range because the model was unbalanced (the levels of frequencies and ranges were unequal). Therefore, the effect of central frequency (category 1) was assessed first, followed by the effect of range (category 2), then by combing categories 3 and 4, the effects of central frequency and range could be assessed independently (and the ANOVA models were balanced). As three ANOVAs were conducted, we reduced the alpha to judge significance accordingly (0.05 divided by 3) to 1.7%.

Only significant main effects and interactions were reported. Pairwise *t*-tests (Bonferroni corrected) were used to follow-up significant effects where there were more than two levels. Cohen’s *d* effect sizes were calculated for the main effects.

When assessing the effects of gender on discomfort, there were significant interactions with central frequency and range. These effects are discussed in Supplementary Figures 3–5.

### 8.2 Results

**FIGURE 9:**
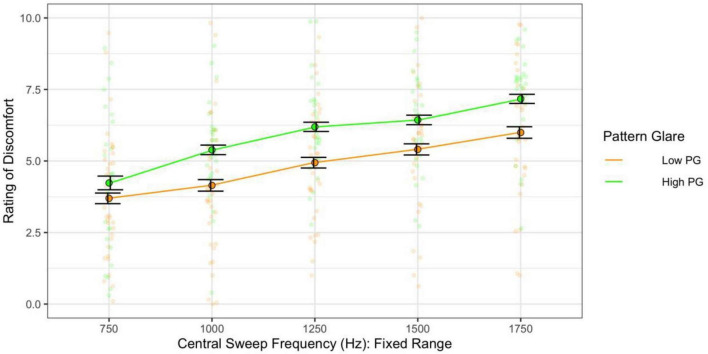
When the frequency range was fixed, higher central frequency still evoked greater discomfort. The high pattern glare (PG) group reported marginally greater discomfort than the low PG group. Error bars show 1 standard error.

Assessing the effect of central frequency alone on discomfort (category 1 sweeps) showed that higher central frequency generated greater discomfort [*F*(4,244) = 37.28, *p* < 0.001; η^2^ = 0.15; *d* < 2.00]. The individuals with high PG reported greater discomfort overall [*F*(1,61) = 5.19, *p* = 0.026; η^2^ = 0.05; *d* = 0.45; not significant with adjusted alpha; Figure 9]. Individuals with migraine, once again, reported greater discomfort, but when compared with headache-free individuals, this comparison was not significant [*F*(1,36) = 3.09, *p* = 0.087; η^2^ = 0.05; *d* = 0.48].

**FIGURE 10:**
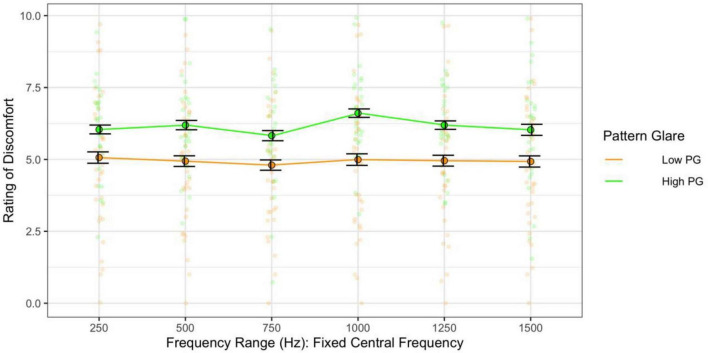
Frequency range, when the central frequency was fixed, had no consistent effect on discomfort. The high PG group reported greater discomfort than the low PG group. Error bars show 1 standard error.

When assessing the effect of the range alone on discomfort (category 2 sweeps), there was no significant effect of range on discomfort [*F*(5,305) = 1.89, *p* = 0.096; η^2^ = 0.004; *d* < 0.58] but those individuals with high pattern glare nevertheless reported all stimuli as more uncomfortable [*F*(1,61) = 6.07, η^2^ = 0.08; *p* = 0.017; *d* = 0.59; Figure 10].

**FIGURE 11:**
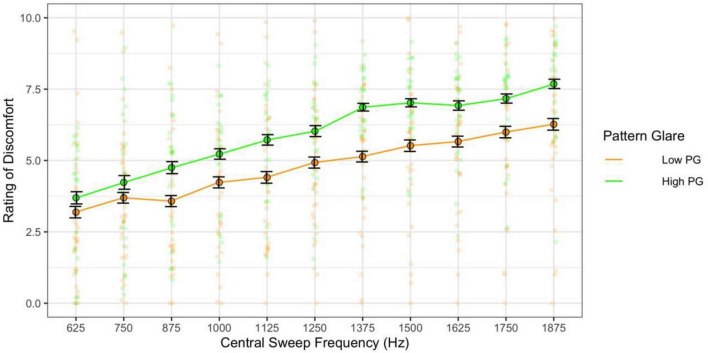
Discomfort increased as a function of central sweep frequency (varying frequency range). Those with high pattern glare (PG) reported greater discomfort overall compared to the low PG group. Error bars show 1 standard error.

**FIGURE 12:**
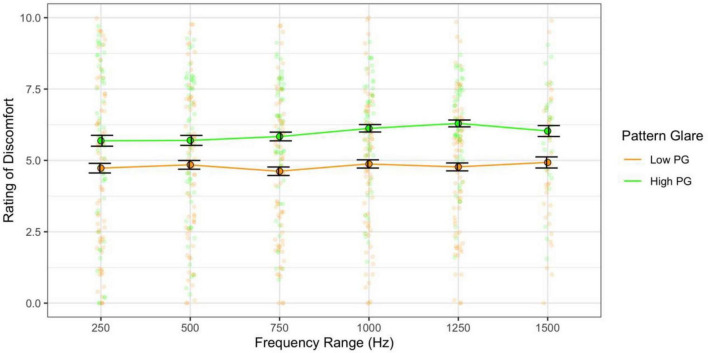
Frequency range had a weak effect on discomfort: the 750 Hz range was nominally more comfortable than other ranges, and the high PG group reported slightly greater discomfort for the larger sweep ranges. Error bars show 1 standard error.

When combining the effects of central frequency and range and assessing their effects independently (categories 3 and 4 sweeps), the higher the central frequency the greater the discomfort [*F*(10,610) = 50.00, *p* < 0.001; η^2^ = 0.20; pairwise *t*-test *p*s < 0.001; *d* < 2.73; Figure 11] and the high pattern glare group once again reported greater discomfort [*F*(1,61) = 7.93, *p* = 0.007; η^2^ = 0.05; *d* = 0.48; Figures 11, 12]. There was also an effect of the range of frequencies used [*F*(5,305) = 3.03, *p* = 0.011; η^2^ = 0.003; *d* < 0.19; Figure 12] because the 750 Hz range was nominally more comfortable than the other ranges (none of the comparisons survived Bonferroni correction). There was also a marginal interaction with PG groups [*F*(5,305) = 2.31, *p* = 0.045; η^2^ = 0.002; not significant with adjusted alpha], where the discomfort for the high PG group was highest for the larger frequency ranges (did not survive *post hoc* comparisons; Figure 12).

## 9 Discussion

The parameters that make sounds uncomfortable to listen to have not been previously identified systematically. Here we focused on frequency and the manner in which it was varied. We also focused on the individual differences that are associated with sensory sensitivity – namely migraine and pattern glare (a measure of visual sensitivity). We found that higher frequencies and rates of sweep, but lower modulation frequencies increased auditory discomfort. The effects of carrier and modulation frequencies on discomfort is consistent with the findings reported by Kumar et al. (2008). The range of frequencies within frequency sweeps had little consistent effect on discomfort. Interestingly, individuals with migraine reported marginally but not significantly greater discomfort compared to headache-free participants. These effects were consistent over several participant samples. However, the effect of visual sensitivity (as defined by the experience of visual illusions, referred to as pattern glare; PG) was consistent across three experiments: individuals with high PG reported greater auditory discomfort than those with low PG. In addition, the estimates of effect size tended to be larger when comparing high and low PG groups compared to migraine and headache-free individuals (see Table 3 for comparison). This suggests that there is a cross-sensory effect such that individuals who are visually sensitive may also have auditory sensitivity.

**TABLE 3:** Cohen’s *d* effect size comparison of migraine and headache-free groups and high and low pattern glare (PG) groups across the four experiments.

Cohen’s *d* comparison	Experiment 1	Experiment 2	Experiment 3	Experiment 4
Migraine vs. headache-free	*d* = 0.19 (29/26)	N/A	*d* = 0.25 (25/13)	*d* = 0.48 (15/22)
High vs. low PG	N/A	*d* = 1.11 (11/8)	*d* = 0.5 (35/28)	*d* = 0.45 (16/36)

The number of migraine and headache-free or high and low pattern glare individuals are shown in parentheses. Experiments 1 and 2 used similar amplitude modulated sounds and so are comparable.

Interestingly, individuals with migraine are just as likely to experience high PG as headache-free individuals (when averaged across Experiments 3 and 4; see Supplementary Table 1). Photophobia, and by proxy, pattern glare, are symptoms of migraine (International Headache Society [IHS], 2018; Wilkins et al., 2021) but are not a fundamental characteristic. Similarly, phonophobia (sensitivity to sound) is also a symptom of migraine but is not a necessary component (International Headache Society [IHS], 2018). Therefore, it appears that we are assessing two different populations when comparing migraine and high PG individuals. This supports the analysis of auditory discomfort in these different populations when identifying individual differences in auditory sensitivity.

Due to the dearth of studies investigating the simple parameters of auditory stimuli that drive discomfort, we can only speculate the mechanisms that drive discomfort in the auditory system. However, in the visual system, visual discomfort is associated with large neural responses. For example, mid-range spatial frequency grating patterns are uncomfortable to view (Wilkins et al., 1984) and neural responses to mid-range spatial frequencies are higher in amplitude than to lower and higher spatial frequencies (Huang et al., 2003; O’Hare, 2017). Similarly, neural responses to chromatic gratings increase with the difference in chromaticity (Haigh et al., 2013, 2015, 2018; 2019). On average, individuals with migraine report greater discomfort (Marcus and Soso, 1989; Haigh et al., 2019) and exhibit larger neural responses than headache-free individuals (Huang et al., 2003; Coutts et al., 2012; Haigh et al., 2019), although this is not always the case (Sharp et al., 2023), highlighting the heterogeneity in migraine sensitivity. Together, these findings suggest that discomfort is linked to cortical excitability.

It is currently unknown whether auditory discomfort is linked to excitability in auditory cortex, and whether individuals with high PG will produce larger auditory neural responses than those with low PG, as we would predict. However, previous models of auditory representation show that low level stimulus parameters are encoded earlier in the auditory stream at the level of the cochlear and brainstem (Shamma, 2003). This model has helped predict the ability to understand speech and discriminate speech from non-speech (Elhilali et al., 2003; Mesgarani and Shamma, 2005). Therefore, it is possible that discomfort may be more closely related to functioning reflected in the auditory brainstem response (ABR). In migraine, there is evidence of abnormal ABRs (Sand and Vingen, 2000; Kochar et al., 2002; Marciszewski et al., 2017) as well as abnormal responses from auditory cortex (Áfra et al., 2000; Ambrosini et al., 2016), but the lack of significant increases in discomfort in migraine suggests that this is not the whole story. Because individuals with high pattern glare report greater auditory discomfort, then assessing their ABRs in addition to their neural responses from primary auditory cortex might illuminate the mechanisms underlying the discomfort.

One point to note is that there is a potential overlap between auditory discomfort and other forms of auditory sensitivity including phonophobia (pain from sound that can occur independently of migraine), misophonia (anger or annoyance to specific stimuli such as chewing, finger tapping, or fidgeting) and hyperacusis (perceiving otherwise innocuous sounds as being loud, painful, or fear-inducing). All three of these conditions tend to affect some individuals more than others and can be debilitating. Note that in Experiment 1, we included a measure of hyperacusis to identify participants with auditory sensitivity independent of migraine, and out of 61 participants, only 2 met the criterion for hyperacusis. This is consistent with previous findings. Estimates of hyperacusis prevalence range between 0.2 and 17.2% (Hannula et al., 2011; Båsjö et al., 2016), with incidence of hyperacusis increasing with age (Andersson et al., 2002). Misophonia is more prevalent and is reported in ∼20% of the American and Chinese undergraduate population (Wu et al., 2014; Zhou et al., 2017) although only 6% reported severe misophonia (Zhou et al., 2017). Prevalence of phonophobia is more difficult to ascertain as it is often reported as a symptom of other conditions such as headache or traumatic brain injury. Due to the relative difficulty in identifying individuals with phonophobia (without a clinical diagnosis) and the low prevalence of misophonia and hyperacusis, we did not focus on these measures of auditory sensitivity. Investigating auditory discomfort in these conditions would be of interest, to see whether there are qualitative and quantitative differences in sound sensitivity.

One of the main limitations of this study is that Experiments 1, 3, and 4 were conducted online on Qualtrics. This meant that we were unable to calibrate the audio systems being used and that the systems varied between individuals. However, when comparing the results from Experiment 1 (online) and Experiment 2 (in-person), the overall results are the same, suggesting that the online format did not significantly impact which parameters drive discomfort. It is likely that the online format added noise to the results making it harder to detect real effects. However, conducting these studies online increases the ecological validity of the studies that the parameters of presentation frequency, sweep frequency, and sweep range likely impact discomfort regardless of the audio system being used. Another potential issue with the study is that ratings of discomfort are highly subjective and up to individual interpretation. Particularly for participants who are less sensitive to auditory stimuli, for example, those who have low pattern glare, they may be basing their ratings on another sensation or parameter of the stimuli. While this is unlikely as there were very few significant interactions between pattern glare and the stimulus parameters across the four experiments, it would be worth exploring the other psychophysical responses to uncomfortable auditory stimuli, such as detection or discrimination thresholds. It is possible that uncomfortable auditory stimuli are easier to detect compared to more comfortable sounds, as seen with uncomfortable visual stimuli (Chronicle et al., 1995). This would help characterize the nature of the auditory sensitivity and could suggest potential signal processing mechanisms underlying the discomfort. A final point to note, is that the number of individuals with migraine or who were headache-free was smaller in Experiment 4, potentially limiting the power needed to detect a significant group difference. However, in Table 3, it is evident that the effect size between migraine and headache-free individuals tended to be smaller than the effect size between high and low PG groups, when compare across the experiments. Experiment 1 had the largest migraine and headache-free groups and yet showed the smallest effect size. For the power analysis, we estimated a medium effect size consistent with the visual discomfort literature. It is possible that the effect size for auditory discomfort is smaller in migraine and so a larger sample size is needed to detect reliable group differences.

The benefits of identifying uncomfortable auditory stimuli are twofold: First, there is some evidence to suggest that reducing the discomfort of the sensory environment improves the ability to process information. For example, visually stressful patterns increased reaction times during a word search task (Allen et al., 2008) and relieving visual discomfort with tinted lenses increased reading speed (Wilkins, 2002; Allen et al., 2008). Examining if the same occurs for conducting tasks while uncomfortable auditory stimuli are present will determine the need to identify and eliminate these stimuli. Second, if sound is to be used to capture attention, for example, for a fire alarm, then using uncomfortable sounds may be helpful for grabbing attention efficiently. Consolidating the statistics of natural sounds may help identify more sounds that are uncomfortable (Monson et al., 2013), just as natural image statistics determine whether images are uncomfortable to view (Fernandez and Wilkins, 2008; Juricevic et al., 2010).

## 10 Conclusion

In summary, we have identified several simple parameters of auditory stimuli that drive discomfort: carrier frequency (pitch), modulation frequency (wobble), and central frequency within sweeps. One of the next steps is to identify these parameters in more complex stimuli that are encountered daily and to compare uncomfortable sounds with the parameters of natural sounds (Monson et al., 2013). Compiling a list of auditory parameters that induce discomfort will help when creating audio media or technology to avoid deterring their target audience. Another future step following this study is to identify the neural response that correlates with discomfort. Understanding the neural mechanisms that are related to auditory discomfort will, in turn, help identify the mechanisms that are related to sensory sensitivity, for example, in individuals with high PG, to target to reduce the discomfort.

## Data availability statement

The datasets presented in this study can be found in online repositories. The names of the repository/repositories and accession number(s) can be found below: https://osf.io/5nymv/.

## Ethics statement

The studies involving humans were approved by the Institutional Review Board at the University of Nevada, Reno (28/01/2019; ID number 1333057). The studies were conducted in accordance with the local legislation and institutional requirements. The participants provided their electronic or written informed consent to participate in this study.

## Author contributions

SH designed the study, analyzed the data, and wrote the manuscript. AH conducted Experiment 2. LM and MM conducted Experiments 1, 3, and 4. All authors reviewed the manuscript.

## Footnotes

1
https://osf.io/5nymv/
