# Supplementary material for: Auditory discomfort in visually sensitive individuals
Source: Front Psychol. 2023 Nov 30;14:1126481. doi: 10.3389/fpsyg.2023.1126481 (PMC10720311; doi:10.3389/fpsyg.2023.1126481)
Supplement: Supplementary file 1 [file Data_Sheet_1.PDF]

Auditory Discomfort in Visually Sensitive Individuals

Sarah M Haigh[1], Anna M Haugland[1], Lourdes R Mendoza[1], Mackenzie Montero[1]

[1]Department of Psychology and Integrative Neuroscience, University of Nevada, Reno

The following contains extended analyses that complement the manuscript. First are the figures from Experiment 2 that illustrated the interaction between carrier and modulation frequency. However, as the same interaction was reported in Experiment 1, the figures are shown here. Second, the number of participants with migraine and high pattern glare (PG) are shown for Experiments 3 and 4. Third,there were effects of gender for Experiment 4 and the interactions are shown here.

Experiment 2

A breakdown of the effects of modulation and carrier frequency on auditory discomfort for all participants are shown in Figure 1 and Figure 2.

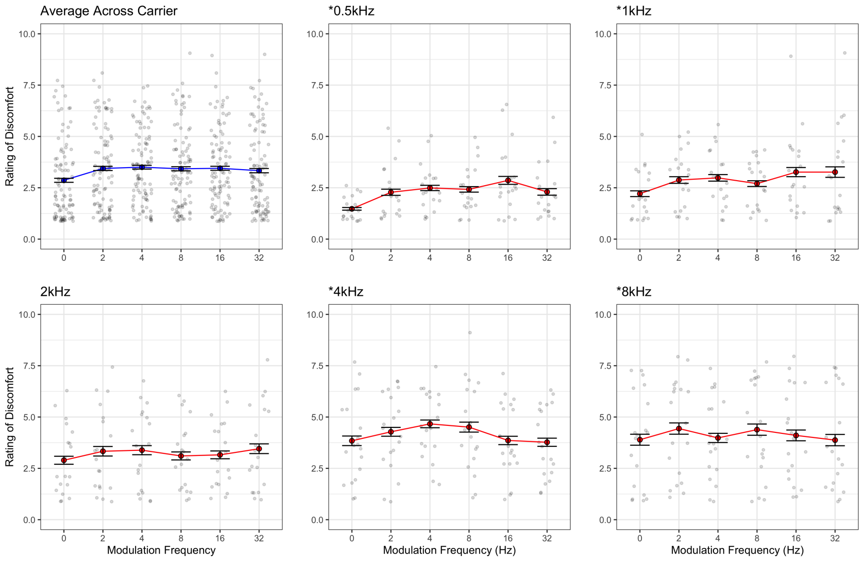

Figure 1: Ratings of discomfort as a function of modulation frequency for each carrier frequency. Error bars show 1 standard error. Note the increase in discomfort as carrier frequency increased. The significant interaction was due to the low carrier frequencies showing increased discomfort with increasing modulation frequencies (0.5kHz and 1kHz), whereas the high carrier frequencies show decreased discomfort with increasing modulation frequencies (4kHz and 8kHz; highlighted by *).

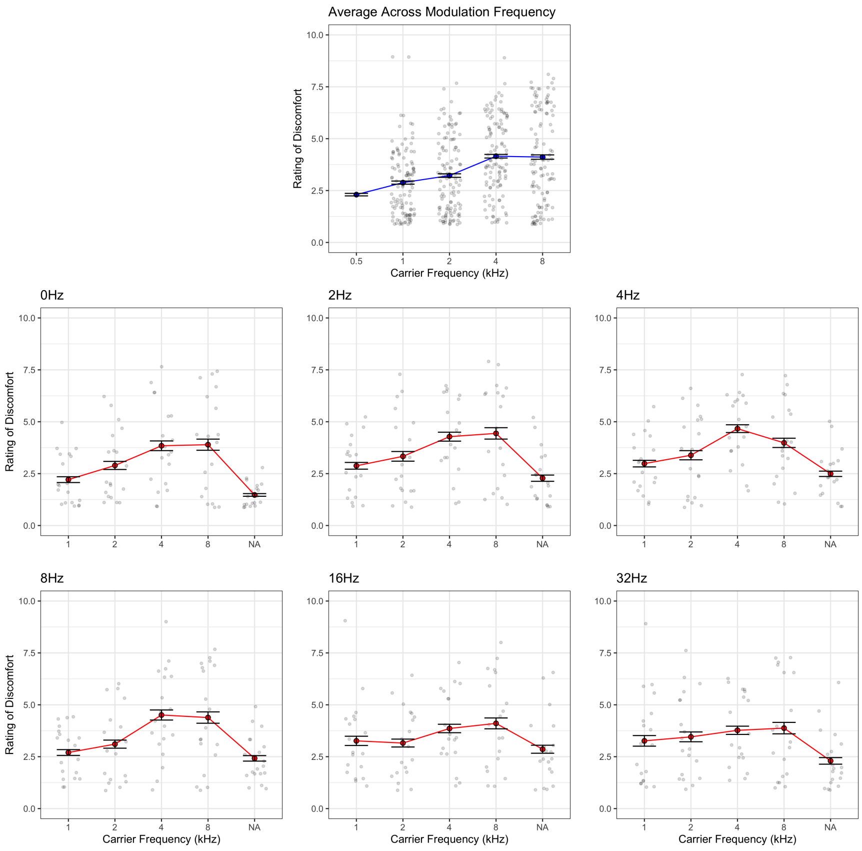

Figure 2: Ratings of discomfort as a function of carrier frequency for each modulation frequency. Error bars show 1 standard error. Note that discomfort increased with carrier frequency for all modulation frequencies. The significant interaction is not evident here.

Migraine and Pattern Glare Groups Overlap

For Experiments 3 and 4, all participants completed the Pattern Glare Test and completed a headache questionnaire about their worst headaches. The headache questionnaire was used to identify participants who had migraines (following IHS, 2018, criteria) and headache-free individuals. Table 1 illustrates the number of participants in each category.

	Migraine		Headache-Free	
	High PG	Low PG	High PG	Low PG
Experiment 3	12 (60%)	8 (40%)	7 (54%)	6 (46%)
Experiment 4	5 (31%)	11 (69%)	17 (77%)	5 (23%)

Table 1: Number of participants in Experiments 3 and 4 who had high or low pattern glare (PG) in the migraine and headache-free groups. Percentages reflect the proportion of migraine or headache-free individuals who experienced high pattern glare (PG) or low pattern glare.

Experiment 4 - Gender Effects

Due to the skewed distribution of males vs females taking part in each of the experiments, gender was included in each of the analyses. However, only Experiment 4 demonstrated effects of gender on ratings of discomfort. The results of including gender as a between-subjects factor in the ANOVAs are presented here.

When assessing the effects of carrier frequency alone, there was a significant interaction between gender and carrier frequency (F(4,232)=5.27, p<.001; η^2=0.02), due to those who identified as female reported greater discomfort to 1750Hz carrier compared to those who identified as male (p=.023, uncorrected for multiple comparisons). No other gender comparisons were significant (Figure 3).

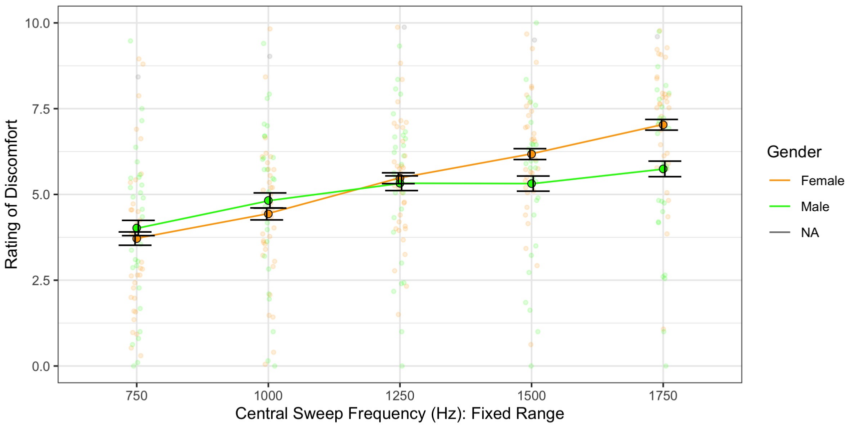

Figure 3: Higher central frequency still evoked greater discomfort, and this was more so for those who identified as female compared to male. Error bars show 1 standard error.

When assessing the effects of range of frequencies alone, there was a significant interaction between gender, pattern glare, and range of frequencies (F(5,290)=2.56, p=.027; η^2=0.005), due to those who identified as female showed significant increases in discomfort in the high compared to low pattern glare groups for the 500Hz and 1000Hz ranges (p=.019 and p=.015 respectively), whereas the males showed effects of pattern glare for the 1000Hz and 1500Hz ranges (p=.025 and p=.034 respectively, uncorrected for multiple comparisons; Figure 4).

Finally, when assessing the effects of central frequency and range of frequencies together, there was once again a significant interaction between gender and carrier frequency (F(10,580)=8.28, p<.001; η^2=0.02), due to those who identified as female showed significant increases in discomfort in the high compared to low pattern glare groups for the 1750Hz and 1875Hz central frequencies (p=.023 and p=.011 respectively, uncorrected for multiple comparisons; Figure 5).

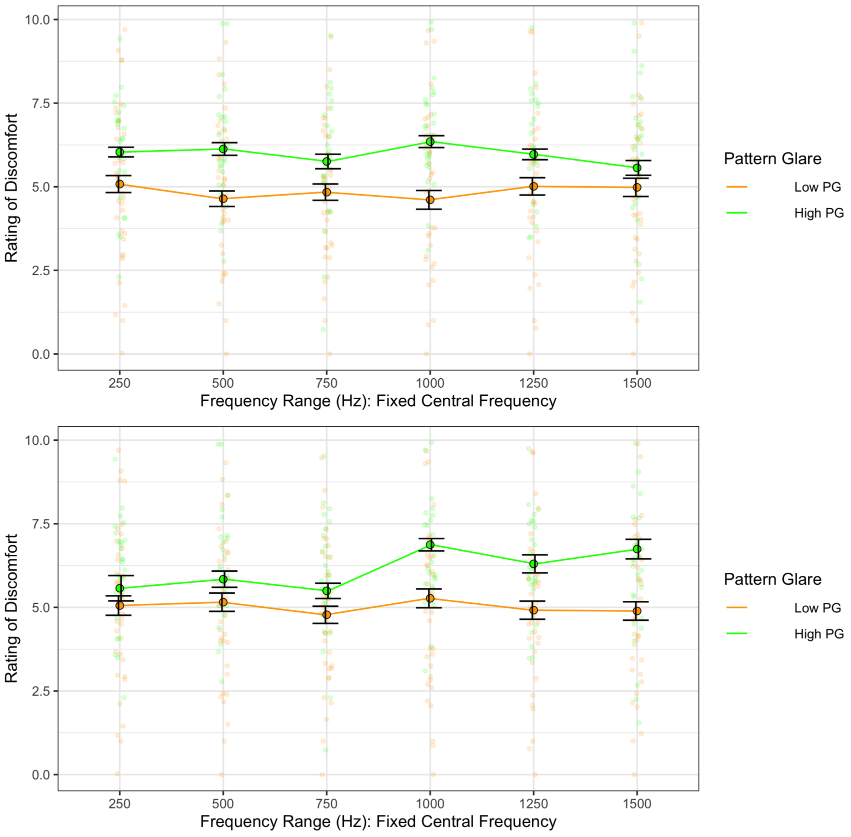

Figure 4: Range increased discomfort overall. Females who had high pattern glare reported greater discomfort for the 500Hz and 1000Hz ranges compared to females with low pattern glare. On the other hand, males with higher pattern glare reported greater discomfort to the 1000Hz and 1500Hz ranges compared to males with low pattern glare. Error bars show 1 standard error.

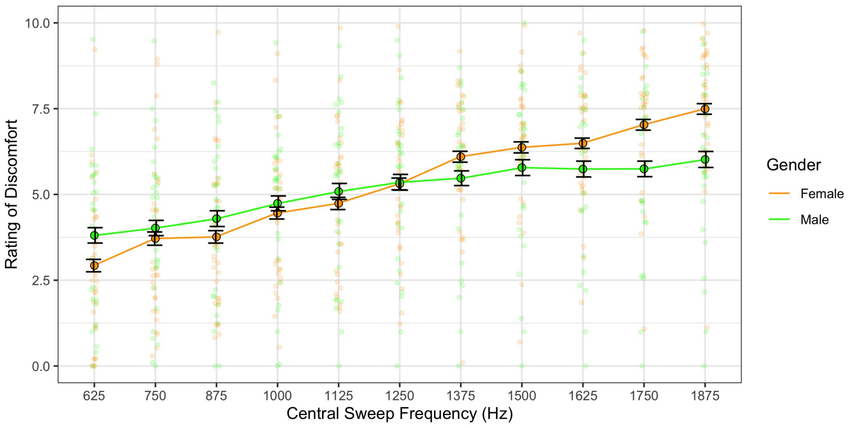

Figure 5: Higher central frequencies still increased discomfort overal. Females reported significantly greater discomfort than males for the 1750Hz and the 1875Hz central frequencies. Error bars show 1 standard error.
